# Transection of the hepatic parenchyma associated or not with the contralateral portal vein branch ligature and its effect in liver regeneration

**DOI:** 10.1590/S1679-45082017AO3831

**Published:** 2017

**Authors:** Henrique de Aguiar Wiederkehr, Julio Cesar Wiederkehr, Luiz Martins Collaço, Eros Luiz de Sousa, Paolo Salvalaggio, Caroline Aragão de Carvalho, Barbara de Aguiar Wiederkehr, Camila Aparecida Moraes Marques, Francielle França da Rosa, Felipe de Negreiros Nanni, Taíse Fuchs

**Affiliations:** 1 Faculdade Evangélica do Paraná, Curitiba, PR, Brazil.; 2 Universidade Federal do Paraná, Curitiba, PR, Brazil.; 3 Hospital Israelita Albert Einstein, São Paulo, SP, Brazil.; 4 Hospital Universitário Evangélico de Curitiba, Curitiba, PR, Brazil.; 5 Hospital Pequeno Príncipe, Curitiba, PR, Brazil.; 6 Pontifícia Universidade Católica do Paraná, Curitiba, PR, Brazil.

**Keywords:** Hepatectomy, Liver regeneration, Swine

## Abstract

**Objective:**

To analyze the influence of portal vein ligation in hepatic regeneration by immunohistochemical criteria.

**Methods:**

Ten pigs divided into two groups of five animals underwent hepatectomy in two stages, and the groups were differentiated by ligation or not of the left portal vein tributary, which is responsible for vascularization of the left lateral and medial lobes of the pig liver. Five days after the procedure, the animals underwent liver biopsies for further analysis of histological and immunohistochemical with marker Ki67.

**Results:**

The group submitted to hepatectomy with vascular ligation showed an increase of approximately 4% of hepatocytes in regeneration status, as well as a greater presence of Kupffer and inflammatory cells as compared to control.

**Conclusion:**

As a result of positive cell replication observed through the Ki67 marker, we can suspect that the ligation of a tributary of the portal vein associated with liver resection promoted a greater stimulus of liver regeneration when compared to liver resection alone.

## RESUMO

**Objetivo:**

Analisar a influência da ligadura da tributária da veia porta no estímulo regenerativo hepático por meio de critérios imuno-histoquímicos.

**Métodos:**

Dez suínos, divididos em dois grupos de cinco animais, foram submetidos à hepatectomia em dois estágios, sendo que os grupos foram diferenciados pela ligadura ou não da tributária da veia porta, responsável pela vascularização dos lobos lateral e medial esquerdos do fígado do suíno. Cinco dias após o procedimento, os animais foram reabordados para retirada de amostras hepáticas para posterior análise de histológica e imunoistoquímica com o marcador Ki67.

**Resultados:**

O grupo submetido à hepatectomia com ligadura vascular apresentou incremento de 4% aproximadamente de hepatócitos em processo de regeneração, bem como grande número de células de Kupffer e células inflamatórias, quando comparado ao controle.

**Conclusão:**

Em virtude da análise positiva da replicação celular observada por meio do marcador Ki67, pode-se observar que a ligadura de uma tributária da veia porta promoveu um maior estímulo de regeneração hepática, efeito observado com menor intensidade no grupo submetido apenas à ressecção hepática.

## INTRODUCTION

The hepatocyte is a highly differentiated epithelial cell that rarely divides. Only one hepatocyte among 20 thousand might be dividing at any one time during the life of a human being or an animal, and this division may occur - at most - once or twice for each cell. Therefore, it is known that one of the characteristics of hepatic tissue is reduced cellular reproduction.^1-3^

Hepatocyte proliferation may be stimulated by different factors, and the destruction of the liver by trauma or infection stands out.^4^ About 30% of normal hepatic parenchyma is compatible with survival and maintenance of liver functions, and up to 20% of the remaining liver is capable of regenerating liver in its space. This recovery results from increased size of the hepatic cells (hypertrophy), along with a rapid cellular division and multiplication (hyperplasia).^4^ These characteristics allow repeated hepatectomies, as long as complications are avoided. During hepatocyte proliferation, there is release of growth factors, such as the hepatocyte growth factor (HGF), transforming growth factor-alpha (∝-TGF), epidermal growth factor (EGF), and fibroblast growth factor (FGF), which determine mitogenic stimulation that affects other liver cells and thus, enables tissue regeneration.^5,6^

Events related to regeneration of hepatic tissue have been described since ancient Greece, where liver regeneration was reported by means of the Prometheus myth, who was condemned to feed an eagle daily with part of his liver. However, during the night, his liver regenerated, providing the bird with eternal food, and Prometheus, with eternal torture.^6^

The first successful experimental model for the hepatic regeneration study was introduced by Higgins and Anderson, in 1931. This model focused on the surgical removal of the left lateral lobe and the medial lobe of rat livers, accounting for approximately 67 to 70% of total hepatic mass of these animals.^7,8^ Since the first liver resection performed by Langenbuch, in 1888, and the first liver transplant performed by Starzl, in 1963, until now, liver surgery has advanced greatly, allowing extensive resections and transplants of parts of the liver to be possible.^9^ Among other factors, this is due to the recognition of the large regenerative capacity of the liver.

Over the last few years, various studies have attempted to describe the initial point in the hepatic regenerative process. Some investigations showed hepatocytes are transferred to the extrahepatic tissue, with synthesis of DNA in the new tissue. These experiments indicate that the mitogenic signs for hepatocytes are systemic, and during this process that occurs during regeneration, the expression of proto-oncogenes related to the cell cycle are noted, not only in the hepatic regeneration process, but in the proliferation of other types of cells as well, and they are immediately activated after hepatectomy. There are some alterations during hepatic regeneration, such as an accumulation of triglycerides, elevation of fetal isoenzymes, and an increase in the enzymatic levels related to DNA synthesis, such as timidine kinase and ornithine decarboxylase.^1^

Ki67 is a protein that acts as a marker of cell proliferation found only during cell division (phases G1, S, G2, and M of the cell cycle). During interphase, the antigen can be detected exclusively inside the nucleus, while in mitosis, most of the protein is dislocated to the surface of the chromosome. This is why it is considered an excellent marker for determining cell growth associated to cancer and other conditions that involve regenerative mechanisms.^9^

Oftentimes, surgical resection is the only potentially curative treatment for patients with primary or metastatic malignant liver disease. However, this resection is limited by the need to preserve a sufficient remnant volume of liver, since an excessive resection can lead to liver failure within a few days after the surgical procedure.^10,11^

To avoid this complication, a future remnant liver (FRL) is recommended, of approximately 25% of total liver volume (TLV), if liver function is normal, and in patients submitted to chemotherapy, the recommendation is for a FRL of approximately 40% of TLV.^12^

The knowledge that the liver remnant volume is a very important limiting factor for the performance of large liver resections led to the creation of new tactics and techniques to prevent postoperative liver failure.^13^ In the beginning of 1990, Makuuchi et al., introduced the technique of portal vein embolization (PVE) as a method of inducing hypertrophy of FRL. Soon afterwards, other authors introduced several techniques that combined portal vein occlusion with staged hepatectomies.^13-17^

A new strategy for patients with marginally resectable liver tumors, previously considered to be unresectable, and originally developed by Hans Schlitt,^19^ was formally reported by Baumgart et al.,^18^ This technique consists of a two-staged hepatectomy with initial portal vein ligation and *in situ* splitting of the liver parenchyma. It is known as Associating Liver Partition and Portal Vein Ligation for Staged Hepatectomy (ALPPS). This technique allows a resection in patients with large or multiple hepatic tumors, that would be of high risk for postoperative liver failure, due to a small FRL.^20^

## OBJECTIVE

To analyze the influence of ligature of the portal vein tributary in the hepatic regenerative stimulus by means of immunohistochemical criteria.

## METHODS

The experimental procedures were performed as per recommendations of the Scientific Committee and the Animal Research Ethics Committee of the *Faculdade Evangélica do Paraná*, Curitiba (PR), approved under number 3893/2014. All animals received care according to the rules of Brazilian legislation for ethics in animal studies (Law 11,794, decree 6,899/2009).

### Surgical procedure

The anesthetic technique used was inhaled general anesthesia using a closed system, and a surgical procedure of two-stage hepatectomy with ligature of the portal vein - ALPPS, was based on the model described by Croome et al.,^20^

The first part of the study involved two pigs and had the objective of verifying if the technique was feasible and could be reproduced. Additionally, we sought to study the hepatic division and vascularization, in order to better plan the surgical procedure.

Pig liver is composed of a right lateral lobe (RL), right medial lobe (RML), left medial lobe (LML), left lateral lobe (LL), and caudate lobe. The pig vena cava is intrahepatic and is located to the right of the liver.

In the initial project, the objective was to use the ALPPS technical standard performed in humans. However, in view of the challenges faced in resecting the parenchyma without causing damage to large vessels, with the intent of performing an extended left hepatectomy, and considering the scarce resources, different from the model, the choice was made to only perform a partial hepatotomy between RML and LML. Hence, they were isolated as deeply as possible, in order to minimize blood loss and the surgical stress caused by an extended operative time.

The second part of the study was divided into two phases. The first phase consisted of performing the equivalent of stage 1 of ALPPS, in which ten animals were submitted to the surgical procedure. The animals submitted to ligature were randomized forming two groups: Group A (pigs 1 to 5), in which the animals were submitted to modified hepatotomy without vascular ligature; and Group B (pigs 6 to 10), in which the animals were submitted to modified hepatotomy with ligature of the portal branch responsible for vascularization of the left lateral and medial lobes of the pig liver.

**Figure 1:**
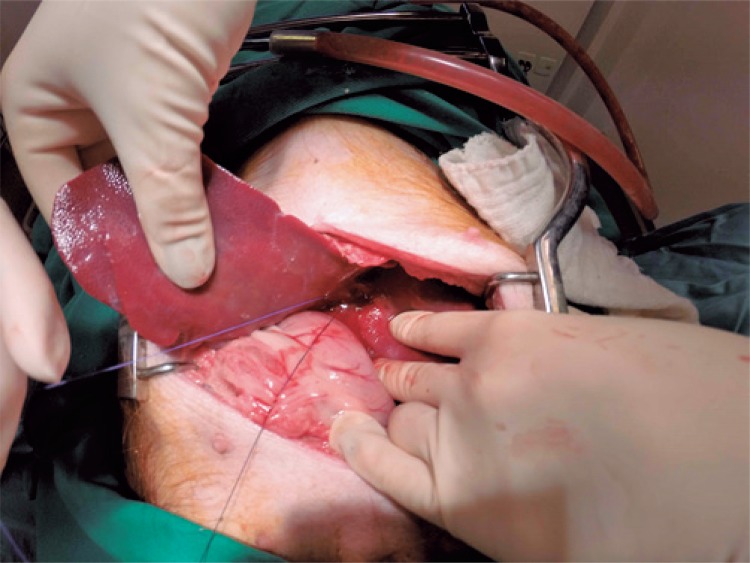
Ligature of the branch of the portal vein responsible for vascularization of the medial and lateral left lobe segments

The vascular ligature was planned based on the illustration of the reference article,^20^ and therefore, the choice was to ligate the left branch of the portal vein, soon after its bifurcation, isolating the blood supply only to lobes LML and LL (Figure 1). Resection of the liver parenchyma was made with the use of an ultrasonic scalpel. Blood and liver parenchyma samples were collected to prepare laboratory parameters and later compare with the postoperative period.

In the second phase, the same animals were used again, five days postoperatively. New liver samples were retrieved for posterior histopathological and immunohistochemical analyses, besides blood for the laboratory panel. After the end of the procedure, the animals were euthanized by means of an anesthetic overdose of thiopenthal, at the dose of 50mg/kg, as per recommendation of the Brazilian guide for good practices for animal euthanasia.

### Biochemical evaluation

Based on the blood sample, a full CBC was done in order to evaluate the counts of platelets, erythrocytes, and leukocytes, using an automated hematologic counter by the flow cytometry method, carried out by an outsourced laboratory (Citolab, Curitiba-PR Brazil).

### Histological evaluation

To perform the histological study, the organs collected were fixated in 10% formaldehyde PBS 0.1M (pH 7.4). Next, fragments were collected for processing according to conventional technique. The organ fragments were included in paraplast and placed so that the slices obtained would result in cross-sectional slices of the organ. Posteriorly, the slices were stained in hematoxylin-eosin and observed on a light microscope, to assess the following parameters: presence of fibrosis, proliferation of bile ducts, presence or absence of inflammatory infiltrate, steatosis, and hydropic degeneration.

To evaluate the quantification of fibrosis, a method was created based on the human fibrosis classification of ISHAK, METAVIR, and the Brazilian Pathology Society (SBP). This scoring system was based on four criteria:

Stage zero: normal/no fibrosis pattern of collagenation.

– Stage 1 or +: fine fibrous septa.

– Stage 2 or ++: wide fibrous septa.

– Stage 3 or +++: wide fibrous septa with penetration into the hepatic parenchyma altering the anatomy.

As to ductal proliferation, the following scoring system was used:

– Normal or +: one to three ducts.

– Moderate or ++: four to six ducts.

– Intense or +++: more than six ducts.

The presence of inflammatory infiltrate, steatosis, and hydropic degeneration was recorded as the presence or absence of these criteria.

The histopathological evaluation was performed by a qualified professional, duly trained and certified in pathology, blinded to the intervention previously made.

### Immunohistochemical evaluation with Ki67

For immunohistochemical analysis, the tissue slices (4µm thick) were embedded in formalin and fixated in paraffin, and submitted to the immunohistochemical technique. This technique consists of the following steps: deparaffinization, rehydration, antigen retrieval, inactivation of endogenous peroxidase, and blockage of unspecific reactions. The primary antibody was incubated for 12 hours at 4**°**C, as per the specific dilution indicated on the package insert of each antibody used. Next, the streptavidin-biotin-peroxidase complex (LSAB^®^, DAKO) was applied; it was developed with diaminobenzidine tetrahydrochloride chromogen (DAB kit, DAKO) and carried out in contrast with 3% hematoxylin. The antibody used was Ki67 (1:200 Santa Cruz Biotechnology, Santa Cruz, CA, USA), assessed in hepatic tissue by pathologists.

Analysis of the slides was based on the immunomarker intensity. The number of hepatocytes marked positively by the Ki67 was determined by manual count in five random visual fields, with a 10x magnification by a professional blinded to the intervention performed.

### Statistical analysis

Means and mean standard error were calculated, and the paired Wilcoxon test was applied. The statistical test was applied for the values obtained in the laboratory tests, with significance of p value p<0.05. To perform the statistical analysis, the GraphPad Prism software, version 14.0 (Statistical Package for Social Science, SPSS Inc., Chicago, IL, USA) was used.

## RESULTS

The statistical evaluation of the values observed for hemoglobin, hematocrit, and platelet count showed no significant difference between the before and after periods in Groups A and B. The statistical analysis of the values obtained for leukocyte count in Groups A and B showed a significant difference, with p values of 0.0061 and 0.0437, respectively (Tables 1 and 2).

**Table 1:** Values obtained in the CBC of Group A (submitted to hepatotomy without vascular ligature)

Variable	Mean	Standard deviation	p value
Hemoglobin (g/dL)			
Before	12.10	1.177	0.6227
After	12.36	1.274	
Hematocrit (%)			
Before	36.40	3.507	0.8843
After	36.00	4.690	
Leukocytes (cells/µL)			
Before	14.290	2.149	0.0061*
After	7.500	1.118	
Platelets (cells/µL)			
Before	46.300	81.210	0.6938
After	479.200	48.849	

* p<0.05.

**Table 2:** Values obtained in the CBC of Group B (submitted to hepatotomy with vascular ligature)

Variable	Mean	Standard deviation	p value
Hemoglobin (g/dL)			
Before	11.16	2.344	0.3879
After	10.24	1.486	
Hematocrit (%)			
Before	33.60	7.021	0.3820
After	30.80	4.550	
Leukocytes (cells/µL)			
Before	8.590	1.746	0.0437*
After	4.930	1.178	
Platelets (cells/µL)			
Before	598.000	43.675	0.1035
After	554.000	78.374	

* p<0.05.

The statistical evaluation of the values obtained for activated prothrombin time (aPTT), serum glutamic pyruvic transaminase (GPT), total bilirubin, and direct bilirubin showed no significant difference before and after in Groups A and B. Analysis of the values obtained for serum glutamic oxalacetic transaminase (GOT) showed no significant difference before and after in Group A, and showed a significant difference of 0.009 in Group B (Tables 2, 3, and 4).

**Table 3:** Values obtained in tests to assess liver function in Group A

Variable	Mean	Standard deviation	p value
aPTT (seconds)			
Before	7.06	0.53	0.0635
After	8.18	0.53	
GOT (U/L)			
Before	60.1	39.290	0.1790
After	31.22	3.263	
GPT (U/L)			
Before	42.90	9.12	0.0945
After	53.66	8.81	
Total bilirubin (mg/dL)			
Before	0.20	0	nc
After	0.20	0	
Direct bilirubin (mg/dL)			
Before	0.1	0	nc
After	0.1	0	

Values before and after show no difference. aPTT: activated prothrombin time; GOT: glutamic oxalacetic transaminase; GPT: glutamic pyruvic transaminase; nc: not calculated.

**Table 4:** Values obtained in tests for evaluation of liver function in Group B

Variable	Mean	Standard deviation	p value
aPTT (seconds)			
Before	7.14	0.71	0.6268
After	7.30	0.45	
GOT (U/L)			
Before	103.1	68.72	0.1674
After	59.56	34.44	
GPT (U/L)			
Before	71.30	11.36	0.009*
After	58.12	7.10	
Total bilirubin (mg/dL)			
Before	0.20	0	0.0993
After	0.28	0.08	
Direct bilirubin (mg/dL)			
Before	0.14	0.05	0.7040
After	0.16	0.09	

* p<0.05. aPTT: activated prothrombin time; GOT: glutamic oxalacetic transaminase; GPT: glutamic pyruvic transaminase.

Another parameter evaluated in the animals submitted to vascular ligature (Group B) was intense vascular congestion. Additionally, in pigs 7 and 9 (Group B), a pattern suggestive of hydropic degeneration was noted (Table 5).

**Table 5:** Histological evaluation of liver tissue of Groups A and B stained with hematoxylin-eosin

Pig	Fibrosis	Ductal proliferation	Inflammatory infiltration	Hepatic steatosis	Hydropic degeneration
1					
Before	+	+	Ø	Ø	Ø
After	+	+	Ø	Ø	Ø
2					
Before	++	+	Present	Ø	Ø
After	+	+	Ø	Ø	Ø
3					
Before	++	+	Ø	Present	Ø
After	++	++	Ø	Ø	Ø
4					
Before	+	+	Ø	Ø	Ø
After	+	+	Ø	Ø	Ø
5					
Before	+	+	Ø	Present	Ø
After	++	++	Present	Ø	Ø
6					
Before	+	+	Ø	Ø	Ø
After	0	+	Ø	Ø	Ø
7					
Before	0	+	Ø	Ø	Ø
After	+	++	Ø	Ø	Present
8					
Before	0	+	Ø	Ø	Ø
After	+	+	Ø	Ø	Ø
9					
Before	+	+	Ø	Ø	Present
After	+	+	Ø	Ø	Ø
10					
Before	0	+	Ø	Ø	Ø
After	+	+	Present	Ø	Ø

Classification of fibrosis: zero, if the collagenation pattern is normal/without fibrosis; + if there are fine fibrous septa; ++ if there are wide fibrous septa. Ductal proliferation classification: + if one to three ducts; ++ if four to six ducts; Ø: absent.

In pigs 3 and 4 (Group A), findings suggestive of liver steatosis prior to the procedure were visualized. The inflammatory infiltrate was observed in only two animals after the procedure, one in Group A and the other in Group B, and it comprised basically monomorphonuclear cells (Table 5).

The results related to proliferation of hepatocytes compared before and after the procedure can be observed on table 6. In Group B there was a significant difference between the number of marked hepatocytes before and after the procedure (p=0.0002).

**Table 6:** Number of hepatocytes marked positively by Ki67, determined by manual count in five random visual fields (10x magnification)

Group	Mean (%)	Standard deviation (%)	p value
A before	2.6	3.578	0.4543
A after	1.2	0.837	
B before	3.8	3.347	0.0002*
B after	7.8	3.347	

* p<0.05.

## DISCUSSION

One of the main stimuli for hepatic regeneration is the resection of the parenchyma, since the postoperative remnant liver has a spectrum of regeneration that is rarely found in healthy livers.

After liver resection, the remnant liver develops and establishes a new state of hyperplasia with a high rate of proliferation of hepatocytes, endothelial cells, and Kupffer cells. These, in turn, play an extremely important role for hepatic regeneration, since from the tumor necrosis factor alpha and interleukin 6, they stimulate the hepatocytes to enter the G1 phase of cellular replication.^21,22^The question that remains is related to what the physiological stimulus would be for this cellular replication.

Some hypotheses, such as the high energy demand generated due to the stress caused by mechanical lesion,^23^ or even an imbalance among the hemodynamic factors,^24^ have been studied and may represent a new paradigm in hepatic regeneration.

Among them, we can highlight a new technique that has been increasingly studied, which consists of hepatotomy in two stages with portal vein ligature, known as ALPPS*.* Described for the first time by Schnitzbauer et al., this technique represented a new alternative for the resection of extensive tumors. It induced hypertrophy and hepatic regeneration with an average of 74% of cases;^25^ these results are superior to those of portal vein ligature techniques or embolization.

Similar results were also reported by Wiederkehr et al.,^10^ in children with large extensive hepatoblastoma, in whom surgical resection represented a high risk of liver failure due to the small size of the FRL. In this case series, the average liver regeneration noted was 72.56%, showing the possibility of a new application of this technique.^10^

The technique, due to its results, raised the hypothesis that redirectioning the vascular flow by ligature of the portal vein in the first stage, without the consequent removal of the specific hepatic lobe, could be an important factor in liver regeneration. Based on this hypothesis, we sought to verify the true role of vascular ligature in liver regeneration.

After analyzing and comparing the rate of hepatic regeneration between the groups, it was noted that Group B, which was submitted to hepatic resection combined with vascular ligature, presented - histologically and in the immunohistochemical analysis with Ki67 - a significant increase of new hepatocytes in the regeneration process, as well as the presence of Kupffer cells and inflammatory cells, a result that is in agreement with that of other articles in which, based on other methods, it was noted that this technique promotes greater hepatic regeneration.^1,19,25^

In this way, one can state that the technique does, indeed, promote a greater stimulus when compared to isolated hepatic resection. This fact is a consequence of the high proliferation rate of hepatocytes and of Kupffer cells, which play a secondary role in liver regeneration.

**Figure 2:**
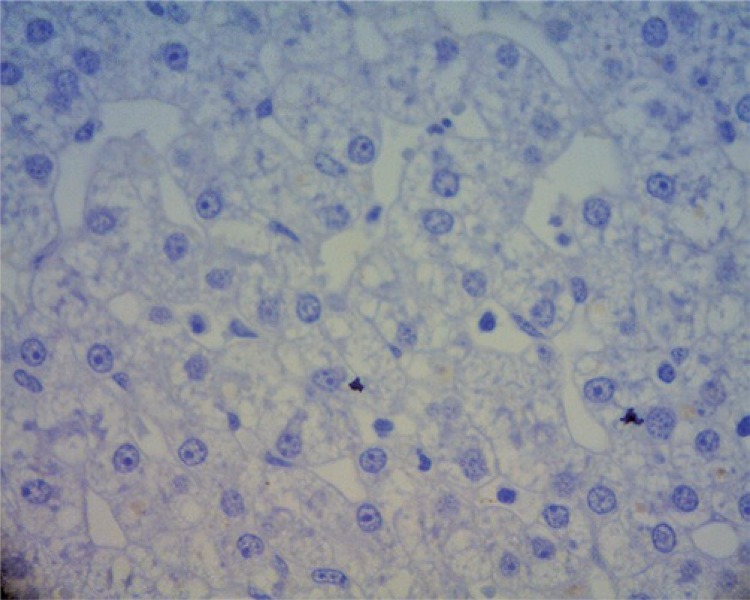
Liver fragment of pig 2 before the surgical procedure (40x magnification)

**Figure 3:**
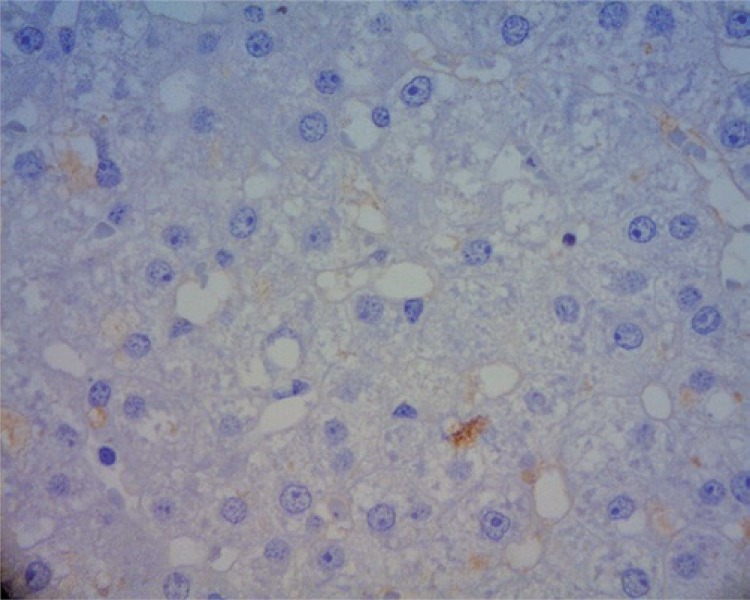
Liver fragment of pig 2, five days after hepatotomy without vascular ligature, showing the absence of nuclei in proliferation (40x magnification)

The explanation for the low rates of hepatic regeneration in Group A (as can be noted in figure 2, corresponds to the liver fragment of pig 2 before the surgical procedure and, in figure 3, it corresponds to the same animal five days after hepatectomy without vascular ligature), can be in the fact that isolated liver resection was not significant enough to stimulate the hepatic regeneration in this region. Due to the anatomy of the pig presenting branching of the intraparenchymatous portal vein, the liver resection became technically more complex, in light of the risk of affecting a vessel of greater caliber with the resection, causing a blood loss of more than the 500mL, the maximum sustained by the animal.

When further correlating these histopathological findings with the laboratory tests, it was not possible to identify great alterations, whether of liver function or even in the coagulogram of both groups, showing safety of the technique used. The increased value of GPT in Group B may be related to the surgical procedure, since it is a nonspecific marker when analyzed separately. GOT is also present in the cells of muscles and of the heart, while GPT is found almost exclusively in liver cells. The latter is, therefore, much more specific for liver diseases than GOT.

The number of leukocytes in Groups A and B showed a statistically significant drop after the procedure. The circulatory redistribution of leukocytes, especially of lymphocytes, is common and generally represents transient responses to a variety of events that cause stress, such as bacterial infections, surgery, trauma, and hemorrhage. These responses can be measured by high levels of endogenous corticoids that induce the drop in circulating lymphocytes, which can be presented as a possible cause of apparent leukopenia in both groups.^26,27^

**Figure 4:**
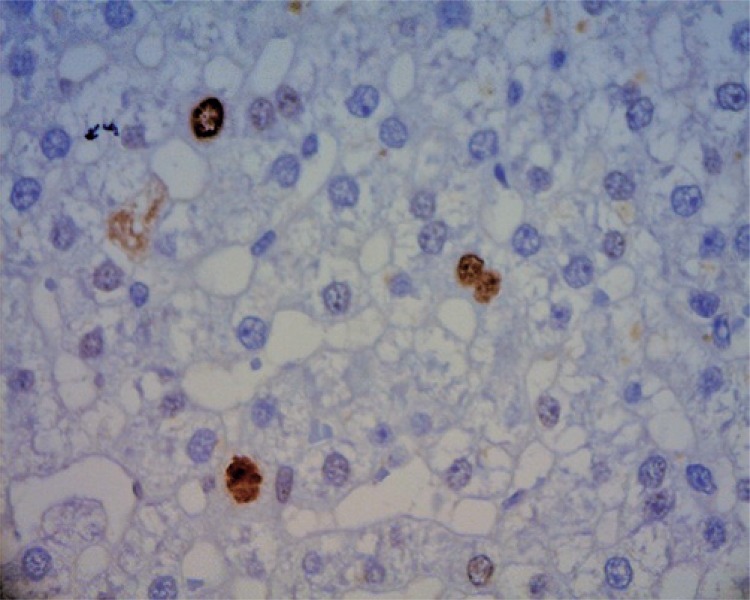
Liver fragment of pig 7, five days after hepatotomy with ligature of the portal branch, showing a large quantity of nuclei marked with Ki67 (40x magnification)

Moreover, considering the hepatic resections were similar in both groups, one can evaluate the ligature of the portal vein branch promoted a greater stimulus of cell regeneration, possibly related to the imbalance of hematologic factors and to redirectioning of blood flow to the contralateral hepatic lobe, as observed on the slide with sample from pig 7, where it is possible, after five days, to note 5% more hepatocytes undergoing regeneration, when compared to the material obtained in the preoperative period (Figure 4).

Croome et al.,^20^described similar results before those of the present study. After the second stage of ALPPS, there was an increase in size of portal spaces in the pigs, as well as in the number of Ki67-positive nuclei, indicating greater cell regeneration, pointing towards the effect of vascular ligature in hepatic regeneration.

## CONCLUSION

This study allowed the establishment of a possible alteration in the two-stage hepatectomy with ligature of the portal vein, since the ligature of only one of its tributaries was performed, maintaining the portal vein pervious and not interfering in the viability and function of the animal liver. Considering the positive analysis of cellular replication by the Ki67 marker, the ligature of a portal vein tributary promoted a stimulation of liver regeneration, an effect not seen in the group submitted to isolated hepatic resection. However, more studies are necessary in order to promote a better understanding of how this event works in liver tissue.

## References

[1] Assy N, Minuk GY (1997). Liver regeneration: methods for monitoring and their applications. J Hepatol.

[2] Chandra S, Mehendale HM (1996). Nutritional modulation of the final outcome of hepatotoxic in injury by energy substrates: an hypothesis for the mechanism. Med Hypotheses.

[3] Fausto N, Laird AD, Webber EM (1995). Liver regeneration. 2. Role of growth factors and cytokines in hepatic regeneration. FASEB J.

[4] Fletcher K, Orton TC, Chipman JK, Strain AJ (1997). The response of hepatocytes isolated from phenobarbitone treated mice to mitogenic growth factors. Arch Toxicol.

[5] Michalopoulos GK, DeFrances MC (1997). Liver regeneration. Science.

[6] Pistoi S, Morello D (1996). Liver regeneration 7. Prometheus’ myth revisited: transgenic mice as a powerful tool to study liver regeneration. FASEB J.

[7] Baratta B, Rizzoli R, Galliani I, Vitale M, Rizzi E, Matteucci A (1996). Early events of liver regeneration in rats: a multiparametric analysis. Histochem Cell Biol.

[8] Yoshida S, Yunoki T, Aoyagi K, Ohta J, Ishibash N, Noake T (1995). Effect of glutamine supplement and hepatectomy on DNA and protein synthesis in the remnant liver. J Surg Res.

[9] Scholzen T, Gerdes J (2000). The Ki-67 protein: from the known and the unknown. J Cell Physiol.

[10] Wiederkehr JC, Avilla SG, Mattos E, Coelho IM, Ledesma JA, Conceição AF (2015). Associating liver partition with portal vein ligation and staged hepatectomy (ALPPS) for the treatment of liver tumors in children. J Pediatr Surg.

[11] Torres OJ, Moraes JM, Lima e Lima NC, Moraes AM (2012). Associating liver partition and portal vein ligation for staged hepatectomy (ALPPS): a new approach in liver resections. Arq Bras Cir Dig.

[12] Shirabe K, Shimada M, Gion T, Hasegawa H, Takenaka K, Utsunomiya T (1999). Postoperative liver failure after major hepatic resection for hepatocellular carcinoma in the modern era with special reference to remnant liver volume. J Am Coll Surg.

[13] Garcea G, Ong SL, Maddern GJ (2009). Predicting liver failure following major hepatectomy. Dig Liver Dis.

[14] Clavien PA, Petrowsky H, DeOliveira ML, Graf R (2007). Strategies for safer liver surgery and partial liver transplantation. N Engl J Med.

[15] Makuuchi M, Thai BL, Takayasu K, Takayama T, Kosuge T, Gunvén P (1990). Preoperative portal embolization to increase safety of major hepatectomy for hilar bile duct carcinoma: a preliminary report. Surgery.

[16] Jaeck D, Oussoultzoglou E, Rosso E, Greget M, Weber JC, Bachellier P (2004). A two-stage hepatectomy procedure combined with portal vein embolization to achieve curative resection for initially unresectable multiple and bilobar colorectal liver metastases. Ann Surg.

[17] Kianmanesh R, Farges O, Abdalla EK, Sauvanet A, Ruszniewski P, Belghiti J (2003). Right portal vein ligation: a new planned two-step all-surgical approach for complete resection of primary gastrointestinal tumors with multiple bilateral liver metastases. J Am Coll Surg.

[18] Baumgart J, Lang S, Lang H (2011). A new method for induction of liver hypertrophy prior to right trisectionectomy: a report of three cases. HPB.

[19] Alvarez FA, Ardiles V, Sanchez Claria R, Pekolj J, de Santibañes E (2013). Associating liver partition and portal vein ligation for staged hepatectomy (ALPPS): tips and tricks. J Gastrointest Surg.

[20] Croome KP, Mao SA, Glorioso JM, Krishna M, Nyberg SL, Nagorney DM (2015). Characterization of a porcine model for associating liver partition and portal vein ligation for a staged hepatectomy. HPB (Oxford).

[21] Chen MF, Hwang TL, Hung CF (1991). Human liver regeneration after major hepatectomy: a study of liver volume by computed tomography. Ann Surg.

[22] Wakabayashi G, Shimazu M, Ueda M, Tanabe M, Kawachi S, Kitajima M (2004). Liver regeneration after resection: molecular and cellular mechanism. Nihon Geka Gakkai Zasshi.

[23] Michalopoulos GK, DeFrances MC (1997). Liver regeneration. Science.

[24] Ibrahim S, Chen CL, Wang CC, Wang SH, Lin CC, Liu YW (2005). Liver regeneration and splenic enlargement in donors after living-donor liver transplantation. World J Surg.

[25] Schnitzbauer AA, Lang SA, Goessmann H, Nadalin S, Baumgart J, Farkas SA (2012). Right portal vein ligation combined with in situ splitting induces rapid left lateral liver lobe hypertrophy enabling 2-staged extended right hepatic resection in small-for-size settings. Ann Surg.

[26] Starzl TE, Marchioro TL, Vonkaulla KN, Hermann G, Brittain RS, Waddell WR (1963). Homotransplantation of the liver in humans. Surg Gynecol Obstet.

[27] Grover C, Bagby Jr, Goldman L, Ausiello D (2008). Leukopenia and leukocytosis. Medicine.

