# *Erratum:* Transection of the hepatic parenchyma associated or not with the contralateral portal vein branch ligature and its effect in liver regeneration

**DOI:** 10.1590/S1679-45082017AO3831E

**Published:** 2017

Henrique de Aguiar Wiederkehr ^1^ ,

Julio Cesar Wiederkehr ^2^ ,

Luiz Martins Collaço ^1^ ,

Eros Luiz de Sousa ^1^ ,

Paolo Salvalaggio ^3^ ,

Caroline Aragão de Carvalho ^1^ ,

Barbara de Aguiar Wiederkehr ^4^ ,

Camila Aparecida Moraes Marques ^2^ ,

Francielle França da Rosa ^5^ ,

Felipe de Negreiros Nanni ^6^ ,

Taíse Fuchs ^1^

^1^ Faculdade Evangélica do Paraná, Curitiba, PR, Brazil.

^2^ Universidade Federal do Paraná, Curitiba, PR, Brazil.

^3^ Hospital Israelita Albert Einstein, São Paulo, SP, Brazil.

^4^ Hospital Universitário Evangélico de Curitiba, Curitiba, PR, Brazil.

^5^ Hospital Pequeno Príncipe, Curitiba, PR, Brazil.

^6^ Pontifícia Universidade Católica do Paraná, Curitiba, PR, Brazil.

In the manuscript “Transection of the hepatic parenchyma associated or not with the contralateral portal vein branch ligature and its effect in liver regeneration”, DOI number 10.1590/S1679-45082017AO3831, published at einstein (São Paulo). 2017;15(2):178-85, there was a spelling error in one of the authors’ surname. The author’s name was corrected from: Paolo Savalaggio to: Paolo Salvalaggio. This error appeared only in the print form of the journal and not in the PubMed database.

